# Multiple nodular and patchy infiltrations in a 34-year-old male

**DOI:** 10.4103/1817-1737.32237

**Published:** 2007

**Authors:** Aysin Sakar, Orhan Temel, Aylin Gulcu, Mine Cabuk, Cihan Goktan, Arzu Yorgancioglu

**Affiliations:** *Celal Bayar University Medical Faculty, Pulmonology, Manisa/Turkey. E-mail: aysins@hotmail.com*; * *Celal Bayar University Medical Faculty, Hematology, Manisa/Turkey*; ** *Celal Bayar University Medical Faculty, Radiology Departments, Manisa/Turkey*

Dear Sir,

All-trans retinoic acid (ATRA), a derivative of vitamin A, is widely used in the treatment of acute promyelocytic leukemia.[1] ATRA syndrome is a treatment complication, including fever, dyspnea, pulmonary infiltrates, pleural/pericardial effusions, episodic hypotension, weight gain and occasionally acute renal failure.[2,3] We are presenting here a case with acute myeloid leukemia who had multiple pulmonary infiltrations and was finally diagnosed as ATRA syndrome after exclusion of other pathologies.

A 34-year-old male presented with fever, cough and dyspnea. He had been diagnosed as acute myeloid leukemia (AML-M3) 2 months ago and was treated by two cycles cytosine-arabinoside (Ara-C) and daunorubicin in addition to ATRA in the second cyclus. His physical examination revealed hypotension of 80/50 mmHg and fever was measured as 39°C. Other system examinations were normal.

A blood sample revealed leucocytosis with leukocyte count of 17,000/ul and anemia with hemoglobin value of 8.5 g/dl. Total platelet count was normal. Blood and urine biochemical tests were normal. Arterial blood gas analysis showed an oxygen saturation of 86% while breathing room air. Cultures of blood and urine were negative for any pathogens.

A chest radiograph was performed, followed by a computed tomography (CT) scan of the chest [Figure 1].

**Figure 1:**
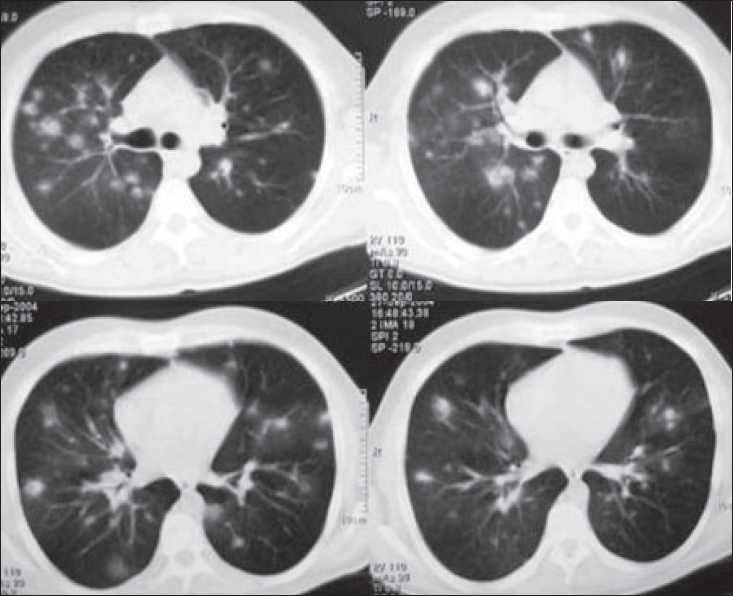
Thorax CT demonstrating multiple patchy and nodular infiltrations

The patient underwent bronchoscopy and bronchoalveolar lavage (BAL). Any significant growth of bacteria, fungus, mycobacteria was not determined in BAL cultures. No atypical cell was detected in pathological examination.

ATRA syndrome was the diagnosis, and dexamethasone was started with 20 mg daily. CT [Figure 2] of the chest on the 10^th^ day of the treatment revealed significant regression in the lesions.

**Figure 2:**
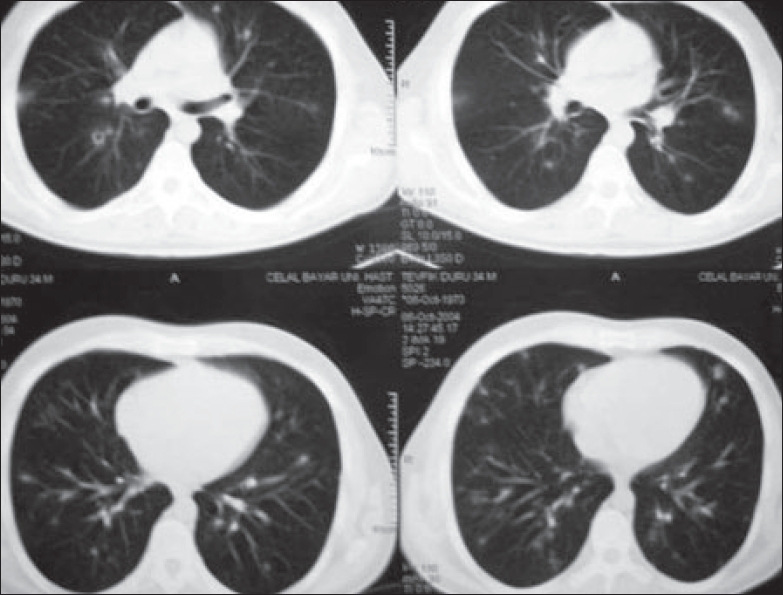
Thorax CT showing regression in the infiltrations

ATRA syndrome is a treatment complication. The risk for the development of ATRA syndrome is reported between 15 and 26%.[2,3] The pathogenesis of the syndrome has not been clearly understood, but it is estimated that it is the result of pathophysiological events caused by ATRA-treated differentiated leukemic cells.[4] These cells infiltrate several organs as a result of their adhesive capacity.[5] Mortality rate is high, ranging between 5 and 30%.[3]

In conclusion, we may state that pulmonary infiltrations may result from pathologies including infections, alveolar hemorrhage, cardiac disorders and pulmonary involvement of underlying diseases in immunocompromised patients. ATRA syndrome should be considered in the differential diagnosis of pulmonary infiltrations in the ATRA-treated patients. Diagnosis is made by excluding other alternatives and the combination of defined symptoms and signs. Early diagnosis and corticosteroid therapy are critical.

## References

[1] Avvisati G, Tallman MS (2003). All-trans retinoic acid in acute promyelocytic leukaemia. Best Pract Res Clin Haematol.

[2] Tallman MS andersen JW, Schiffer CA, Appelbaum FR, Feusner JH, Ogden A (2000). Clinical description of 44 patients with acute promyelocytic leukemia who developed the retinoic acid syndrome. Blood.

[3] De Botton S, Dombret H, Sanz M, Miguel JS, Caillot D, Zittoun R (1998). Incidence, clinical features, and outcome of all trans-retinoic acid syndrome in 413 cases of newly diagnosed acute promyelocytic leukemia. The European APL Group. Blood.

[4] Larson LS, Tallman MS (2003). Retinoic acid syndrome: Manifestations, pathogenesis and treatment. Best Pract Res Clin Haematol.

[5] Frankel SR, Eardley A, Lauwers G, Weiss M, Warrell RP (1992). The retinoic acid syndrome in acute promyelocytic leukemia. Ann Intern Med.

